# Electrical stimulation of the superior colliculus induces non-topographically organized perturbation of reaching movements in cats

**DOI:** 10.3389/fnsys.2015.00109

**Published:** 2015-07-28

**Authors:** Jean-Hubert Courjon, Alexandre Zénon, Gilles Clément, Christian Urquizar, Etienne Olivier, Denis Pélisson

**Affiliations:** ^1^Integrative, Multisensory, Perception Action and Cognition Team, Centre de Recherches en Neurosciences de Lyon, INSERM U1028 and CNRS UMR5292, BronFrance; ^2^Institute of Neuroscience, Université Catholique de Louvain, BrusselsBelgium

**Keywords:** forelimb movement, motor map, electrical stimulation

## Abstract

Besides its well-known contribution to orienting behaviors, the superior colliculus (SC) might also play a role in controlling visually guided reaching movements. This view has been inferred from studies in monkeys showing that some tectal cells located in the deep layers are active prior to reaching movements; it was corroborated by functional imaging studies performed in humans. Likewise, our group has already demonstrated that, in cats, SC electrical stimulation can modify the trajectory of goal-directed forelimb movements without necessarily affecting the gaze position. However, as in monkeys, we could not establish any congruence between the usual retinotopic SC map and direction of evoked forelimb movements, albeit only a small portion of the collicular map was investigated. Therefore, the aim of the current study was to further ascertain the causal contribution of SC to reaching movement by exploring the whole collicular map. Our results confirmed that SC electrical stimulation deflected the trajectory of reaching movements, but this deviation was always directed downward and backward, irrespective of the location of the stimulation site. The lack of a complete map of reach directions in the SC and the absence of congruence between the direction of evoked forelimb movements and the collicular oculomotor map challenge the view that, in the cat, the SC causally contributes to coding forelimb movements. Interestingly, the very short latencies of the effect argue also against the interruption of reaching movements being driven by a disruption of the early visual processing. Our results rather suggest that the SC might contribute to the reach target selection process. Alternatively, SC stimulation might have triggered a postural adjustment anticipating an upcoming orienting reaction, leading to an interruption of the on-going reaching movement.

## Introduction

The superior colliculus (SC) is a layered midbrain structure characterized by topographically organized sensory and oculomotor maps (Wurtz and Albano, 1980; May, 2006), known to be involved in orienting behaviors such as saccadic eye movements (Wurtz and Albano, 1980; Sparks and Hartwich-Young, 1989; Sparks, 2002), head movements (Olivier et al., 1995; Corneil et al., 2002; Walton et al., 2007), and gaze shifts (Guitton, 1992; Freedman, 2008). Other higher-level processes have also been assigned to the SC, including motor preparation (Dorris et al., 1997, 2007; Dorris and Munoz, 1998), target selection (McPeek and Keller, 2002; Krauzlis et al., 2004), and attention allocation (Kustov and Robinson, 1996; Ignashchenkova et al., 2004; Zénon and Krauzlis, 2012; Krauzlis et al., 2013).

During the past two decades, several electrophysiological studies in monkeys have suggested a possible contribution of the SC to the control of goal-directed arm movements (Werner, 1993; Werner et al., 1997a; Stuphorn et al., 1999, 2000). Earlier electrophysiological and anatomical studies had already hinted such an interaction between the SC and forelimb control (Anderson et al., 1972; Grantyn and Grantyn, 1982). The causal contribution of the SC to arm movement control was further documented by studies performed in monkeys showing that SC electrical stimulation induced arm movements (Philipp and Hoffmann, 2014) and by human functional imaging studies having identified a reach-related activation in the SC contralateral to the moving arm (Linzenbold and Himmelbach, 2012; Himmelbach et al., 2013). Altogether, these results are presented as evidence that the SC is part of a subcortical circuit which participates in visually guided arm movement initiation and/or execution, and which permits rapid corrections of on-going reaching movements (Philipp and Hoffmann, 2014).

Despite this accumulating body of evidence, the exact role played by the SC in encoding reaching forelimb movements remains unclear, since, amongst other things, a clear congruence between the well-known retinotopic SC map and the topography of the arm-related cells, or of the effect of electrical stimulation, has never clearly been demonstrated (Werner et al., 1997a; Philipp and Hoffmann, 2014). Some authors have even suggested that the SC might contribute to reaching through an effector independent process, such as target selection (Song et al., 2011; Song and McPeek, 2015).

In a previous study, we aimed at searching for a causal role of the SC to reaching movement in cats, a species in which the SC is expected, from a phylogenetic perspective, to have a stronger influence on spinal circuitry (Nudo and Masterton, 1989; Olivier et al., 1991). We found that, in animals trained in reaching a piece of food with their right paw, electrical stimulation applied into the deep collicular layers at the onset of forelimb movements led to a significant deceleration and downward deflection of these movements (Courjon et al., 2004). Because gaze shift was not systematically elicited by SC stimulation, this finding was seen as additional evidence that the cat’s SC is causally involved in controlling visually guided forelimb movements. However, because only a restricted region of the SC was explored, once again, a clear congruence between the SC retinotopic map and electrically evoked forelimb deviations could not be established. As a consequence, we did not have a chance to definitively determine the functional significance to these findings.

Hence, the aim of the current study was to determine whether such a congruence exists, and to do so, we applied electrical stimulation to a larger area of the collicular oculomotor map. The results confirm that, in cats, SC electrical stimulation perturbs visually guided reaching movements, but further reveal that the deflection of the on-going movement does not depend on the location of the stimulation electrode on the SC retinotopic map.

## Materials and Methods

All experimental procedures were conducted in accordance with the guidelines of the French Ministry of Agriculture (87/848) and the European Community (86/609/EEC). The animals were housed and cared for in accordance with these guidelines. Three cats were used in the current study; the results from cat Y have already been published previously in a preliminary report (Courjon et al., 2004).

### Experimental Set-Up

**FIGURE 1:**
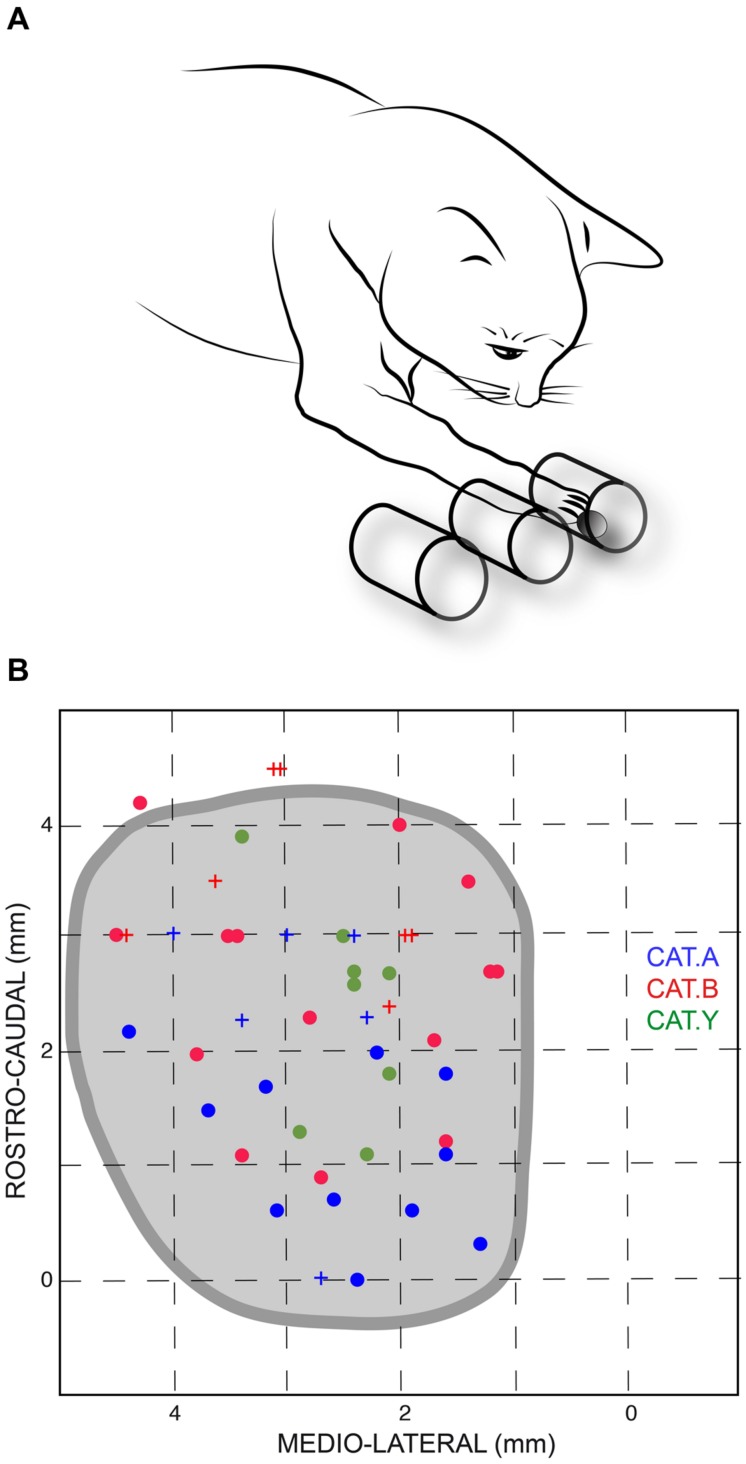
**(A)** Sketch of the experimental setup. Cats were trained to reach a piece of food located randomly in one of the three horizontal tubes placed in front of them; the leftmost tube was aligned with the sagittal axis of the animal. **(B)** Projections of stimulation site locations for the three cats and outline of the left superior colliculus (SC) drawn from horizontal sections of the stereotaxic atlas of the cat’s brainstem (Berman, 1968). The horizontal and vertical axes represent the stereotaxic coordinates, expressed in mm, along the medial-lateral and rostral-caudal axes, respectively. Sites from which perturbations of the reaching movements were consistently elicited are indicated by dots; crosses show ineffective stimulation sites for limb movements. Each cat is identified by a unique color.

The animals were mildly restrained by a harness and were trained to stand still, on four legs, in front of three parallel open tubes placed horizontally at their shoulder level. The cats were trained to reach, with their right paw, a piece of food located in one of these tubes. The leftmost tube was aligned with the sagittal axis of the animal; the left, middle, and right tubes corresponded to movement direction of 0, 28 and 45°, respectively, with respect to the cat’s sagittal axis (**Figure 1A**). The internal diameter of the tubes was 30 mm and their inter-axis distance was 35 mm.

Vertical and horizontal gaze and head position signals were measured using the search coil method and sampled at 500 Hz (FIR filter, 70 Hz cut-off frequency) and stored on a PC for oﬄine analysis. The position of the right forelimb extremity was measured by a method detailed in a previous paper (Urquizar and Pélisson, 1992). In brief, the 3D coordinates of a pair of infrared LEDs fixed on the cat’s wrist were monitored by two orthogonally mounted 2D sensors (Hamamatsu, spatial resolution: 0.1 mm, sampling rate: 324 Hz), displayed on a computer monitor and stored on a PC for off-line analysis.

When the training was completed, the animals were anesthetized by pentobarbital sodium (I.P. injection 30 mg/kg; I.V. perfusion: 1–3 mg/kg/h) and implanted with an eye coil to allow us to record gaze movements (Guillaume and Pélisson, 2001). Then, the skull was exposed, a craniotomy was made, and a chamber was implanted over the midline to allow access to both SC with microelectrodes. Finally, a plastic post was inserted in the acrylic headpiece to restrain head movements when necessary (see below). Another coil was also embedded into the headpiece to record head movements. Then the cats underwent a 2-week recovery period during which they received antibiotics and their wounds and recording chamber were cleaned daily using antiseptic agents.

### SC Stimulation

At the beginning of each experiment, the head of the animal was kept restrained while a tungsten microelectrode was lowered into the left SC by means of a lightweight ultra compact micromanipulator (Narishige, MO-903) attached to the recording chamber. The SC surface was identified by the typical visual responses recorded when the electrode entered the superficial layers. Then, the electrode was lowered by 1.3–2.6 mm below the SC surface, corresponding to the depth where electrical stimulation elicits eye movements (Guillaume and Pélisson, 2001). Finally the head of the animal was released for the remaining of the experimental session, which lasted for about 1 h.

Before investigating the effects of SC electrical stimulation on reaching movements, we first determined the threshold of electrically evoked gaze shifts for each stimulation site. To do so, 300 ms trains of 0.5 ms pulses were delivered at 300 Hz at various intensities (Guillaume and Pélisson, 2001). The minimal current intensity required to evoke a gaze shift with a probability of about 75% was defined as the gaze shift threshold (GST). Then, the stimulating electrode was kept at the same location for the remainder of the session. During the first reaching movements performed by the cats, we searched for the appropriate stimulation parameters to perturb forelimb movements reliably. To do so, the train duration was first reduced to 70–200 ms, which was previously found to be sufficient to alter the on-going forelimb movements (Courjon et al., 2004). Second, the stimulation intensity was then slightly increased to compensate for the shorter train duration by steps of 5 μA until a noticeable forelimb perturbation was observed. The forelimb perturbation threshold (FPT) was defined as the minimal current intensity required for altering the forelimb movement in at least 75% of the trials, as assessed by visual inspection of the movement trajectory displayed after each trial on a computer monitor. If no movement perturbation was detected for a 7 × GST intensity, the session was stopped and this site was discarded. The intensity used for all limb movements perturbations was on average close to the FPT value, corresponding to 2.6 × GST (±1.4), 3 × GST (±1.9), and 1.4 × GST (±0.5) for cats A, B, and Y, respectively.

Once the intensity of the SC stimulation required to perturb forelimb movements was determined, microstimulations were triggered automatically by using a movement velocity threshold. Stimulations were elicited when the forelimb velocity along the *X*-axis (depth) was on average 265 mm/s for the three cats. Trials during which an electrical stimulation was delivered were interleaved randomly (probability of 33%) with trials without electrical stimulation. The whole experimental session was videotaped at a frequency of 25 images/sec and synchronized with the recording of the 3D coordinates of the cat’s wrist on the computer.

### Data Acquisition and Analysis

Signal processing and analyses were performed off-line with a custom-made software running in MATLAB^®^. A velocity vector of forelimb displacements was computed from the 3D coordinates (X: depth, Y: azimuth, Z: elevation) of the forelimb position. The onset of reaching movements was determined by using a velocity threshold of 50 mm/s. The offset was defined as the time when the paw entered the target tube, as determined during the off-line analysis of the videotape.

**FIGURE 2:**
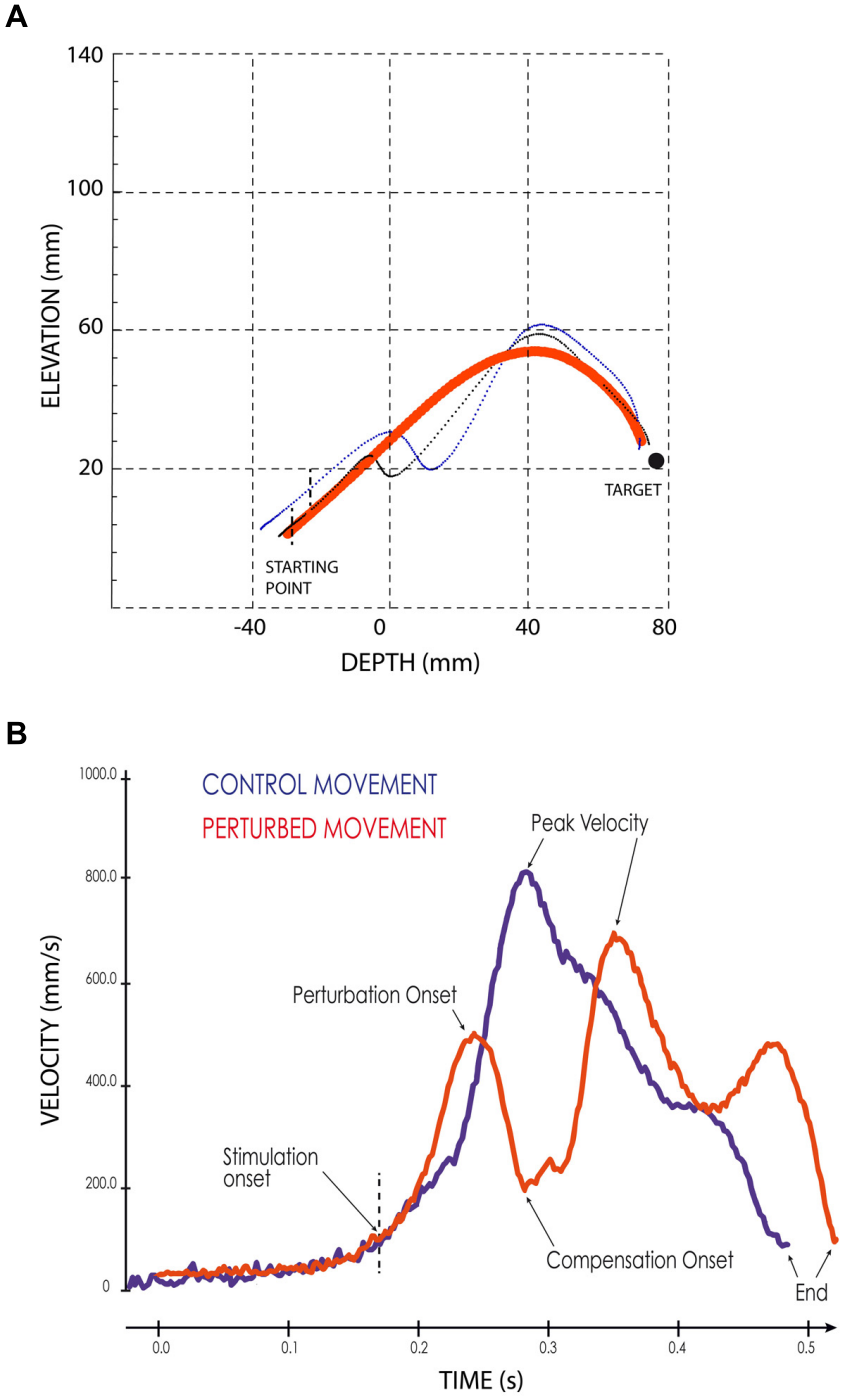
**Examples of perturbed reaching movements.** **(A)** Lateral view of two typical forelimb movements following SC electrical stimulation (thin black and blue lines) superimposed on the average path of four control movements (thick red line). Black vertical dashed lines indicate the stimulation onset. The black filled circle symbolizes the target. **(B)** Velocity profiles of a control (blue line) and of a perturbed (red line) forelimb movements. The arrows indicate the different temporal parameters; the vertical dashed line indicates the onset of the electrical stimulation.

When the SC stimulation elicited a forelimb movement deflection, we first determined the perturbation onset, defined visually from the movement velocity time-course as the time when the continuous increase of forelimb velocity reverted to a velocity decrease, i.e., when the forelimb started to decelerate (see **Figure 2B**). Second, we measured the onset of the movement correction that followed consistently the stimulation-induced perturbations. This onset was defined as the next velocity reversal, i.e., when the limb accelerated again following the stimulation offset (see **Figure 2B**). The effect of electrical stimulation on reaching movements was quantified by computing a “perturbation vector” in 3D space for each session and each of the three different cats. This vector was obtained by calculating the largest difference between the path of a particular perturbed movement and the average path of control movements performed toward the same target (Courjon et al., 2004). Finally, the amplitude of the forelimb movement perturbation was calculated as the norm of this vector (Courjon et al., 2004).

## Results

The current results confirmed our previous finding that electrical stimulation of the deep layers of the cat’s SC deviates on-going forelimb reaching movements (Courjon et al., 2004). Here we explored a larger portion of the SC to search for a possible correspondence between the SC motor map and the direction and/or amplitude of the perturbation vector. The distribution of stimulation sites for the three cats is illustrated in **Figure 1B**; the explored zone represented a rather large portion of the SC surface, spanning about 4 mm along the rostro-caudal and 3 mm along the medial-lateral axis. The GST was determined for each stimulation site from which a forelimb perturbation could be elicited (dots in **Figure 1B**) and ranged from 4 to 40 μA, with a mean value of 12.4 ± 5.2 μA (*n* = 11) for cat A, 14.9 ± 11.6 μA (*n* = 15) for cat B, and 16.5 ± 9.1 μA (*n* = 11) for cat Y. These GST values are comparable to those reported in the literature (Guillaume and Pélisson, 2001).

Thirty-seven out of the 49 stimulation sites (75.5%) explored in the three cats led to a perturbation of the reaching movements. We investigated whether the possibility to evoke motor responses varied with the position of the stimulating electrode on the SC oculomotor map. First, concerning the evoked gaze shifts, we found that the GST was significantly correlated with the medial-lateral location of the stimulated site (*r* = 0.34, *p* = 0.019), indicating that GST was significantly larger for stimulation sites located more medially. Second, concerning the perturbation of forelimb movements, the FPT was generally higher for stimulation sites located more medially, and the correlation between FPT and the medial-lateral site position almost reached statistical significance (*r* = 0.44, *p* = 0.06). In addition, there was no correlation between the rostral-caudal coordinate of the site position and either FPT (*r* = 0.12, *p* = 0.49) or GST (*r* = 0.02, *p* = 0.90). Finally, there was a significant correlation between FPT and GST (*r* = 0.74, *p* = 0.01). Overall, these results indicate that lateral parts of the cat colliculus have stronger sensitivity to electrical stimulation. Note also that, in about half of the trials (49, 71, and 40% in cats A, B, and Y, respectively), no associated gaze displacement accompanied the forelimb perturbation and, when present, they were of small amplitude, as already reported in our previous study (Courjon et al., 2004).

As illustrated in **Figure 2A**, electrical stimulation of the SC induced a clear inflection in the path of the reaching movements. This perturbation consisted in a reversal of the instantaneous movement acceleration (**Figure 2B**) leading to a transient braking of the on-going movement. As the stimulation was elicited during the acceleration phase of the forelimb movement, the velocity measured at the time of this reversal represented only a fraction (about 70%, see **Table 1**) of the normal peak velocity reached during unperturbed forelimb movements.

**Table 1:** Descriptive statistics (Mean and SD) for the three cats of threshold values (gaze and limb movements), control movement parameters and electrically induced perturbations.

		Threshold		Control movements		Perturbed movements			
Cats	Number of sessions	Gaze displacement (μA)	Limb movement (μA)	Peak velocity (mm s^-1^)	Duration (ms)	Peak velocity (mm s^-1^)	Duration (ms)	Perturbation latency (ms)	Perturbation amplitude (mm)
A	11	12.4(5.2)	34.4(15.3)	1590.7(184.3)	173.4(24.3)	1174.6(307.8)	260.0(59.9)	37.6(6.9)	18.1(6.8)
B	15	14.9(11.6)	42.1(32.3)	985.3(125.3)	236.3(27.3)	724.7(82.9)	278.1(34.5)	49.1(13.0)	12.1(3.1)
Y	11	16.5(9.1)	24.0(13.1)	838.8(156.4)	304.5(70.4)	537.1(136.8)	430.0(110.3)	56.8(10.9)	27.5(6.5)

**FIGURE 3:**
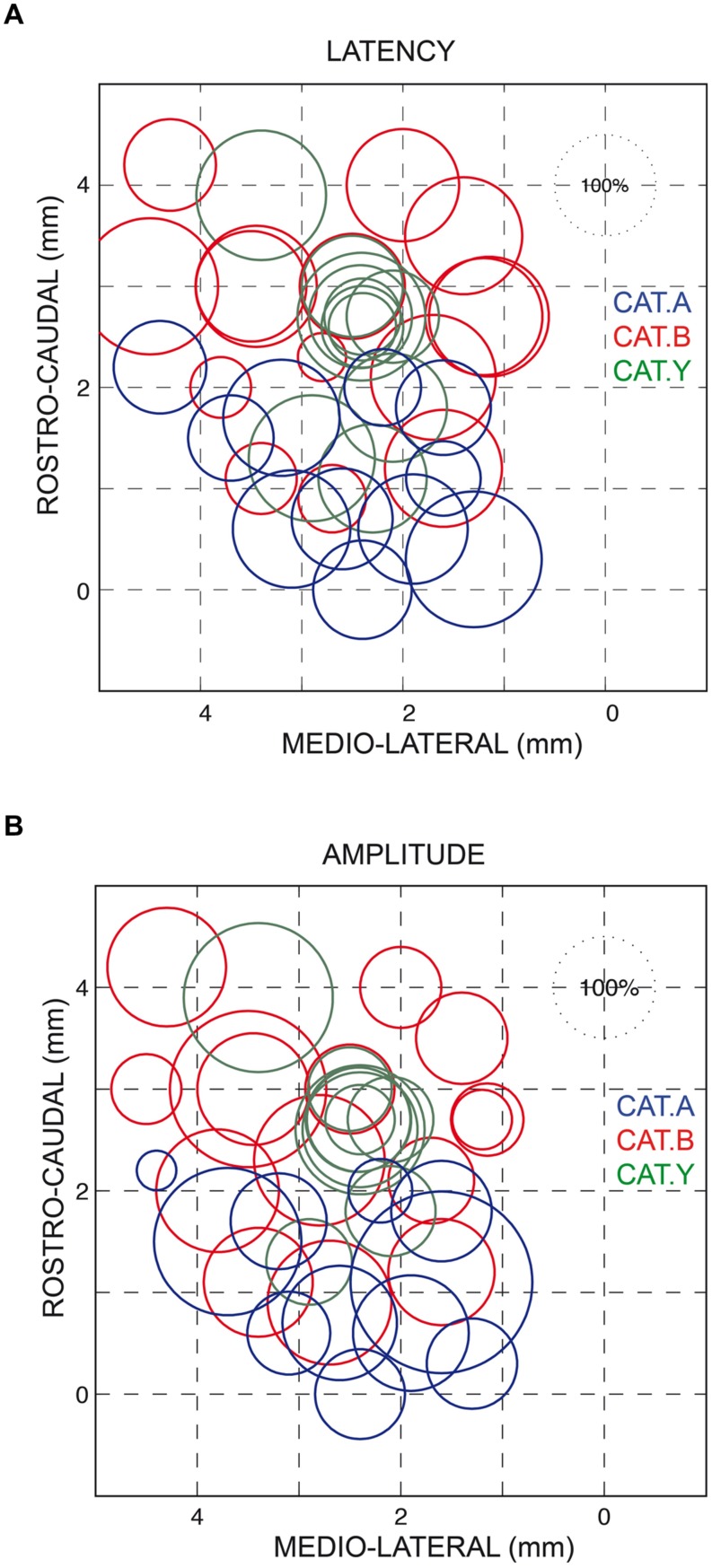
**Normalized latencies **(A)** and amplitudes **(B)** of the forelimb movement perturbations following SC electrical stimulation plotted on a dorsal view of the left SC.** The diameter of the circles represents the latency/amplitude of the perturbation expressed as a percentage of the average value computed for all three cats (100% circle in the upper right corner of each figure). The horizontal and vertical axes represent the stereotaxic coordinates, expressed in mm, along the medial-lateral and rostral-caudal axes, respectively. Each cat is identified by the same color as in **Figure 1**.

As also shown in **Figure 2A**, all forelimb movements resumed after stimulation to reach the original goal with normal accuracy. Reaching errors, i.e., trials in which the paw of the animal failed to enter the target tube, were never observed in those experiments. As a consequence of the electrically induced perturbation, the overall duration of the reaching movements increased in all three cats (see **Table 1**). The latency of forelimb perturbation (measured as the delay between the onset of electrical stimulation and perturbation onset) was 37.6 ± 6.9 ms for cat A, 49.1 ± 13.0 ms for cat B, and 56.8 ± 10.9 ms for cat Y; this difference between animals was significant [ANOVA, *F*(2,34) = 8.7; *p* < 0.001]. The perturbation latency for each individual site was normalized with respect to the mean perturbation latency obtained in a given animal (**Figure 3A**), allowing us to compute a correlation between the normalized latencies (LAT), expressed in percentage of the mean, and the location of the stimulation sites on the SC map (X and Y for medio-lateral and rostro-caudal coordinates, respectively). These correlations between the normalized latencies and the medio-lateral and rostro-caudal location of stimulation sites were not significant (*r* = -0.15, *p* = 0.38; *r* = 0.103, *p* = 0.54, respectively). These negative results were confirmed by a multiple regression approach in which LAT = 105.2 + 2.83^∗^X – 4.32^∗^Y (*r* = 0.20, *p* = 0.50).

Similarly, we found no correlation between the amplitude of the perturbation vector (AMP) and the stimulation site location on the SC map (medio-lateral axis: *r* = 0.13, *p* = 0.46; rostro-caudal axis: *r* = 0.06, *p* = 0.70, see **Figure 3B**). This finding was corroborated by the non-significant result of the multiple regression approach (AMP = 93.6 – 2.45^∗^X + 4.56^∗^Y; *r* = 0.15, *p* = 0.67).

We also found no significant correlation between AMP and LAT (*r* = 0.31, *p* = 0.65). Only a significant negative correlation was found between AMP and the normalized values of both FPT (*r* = -0.34, *p* = 0.039) and GST (*r* = -0.39, *p* = 0.02), indicating that the lower the FPT and GST, the larger the induced forelimb perturbation.

**FIGURE 4:**
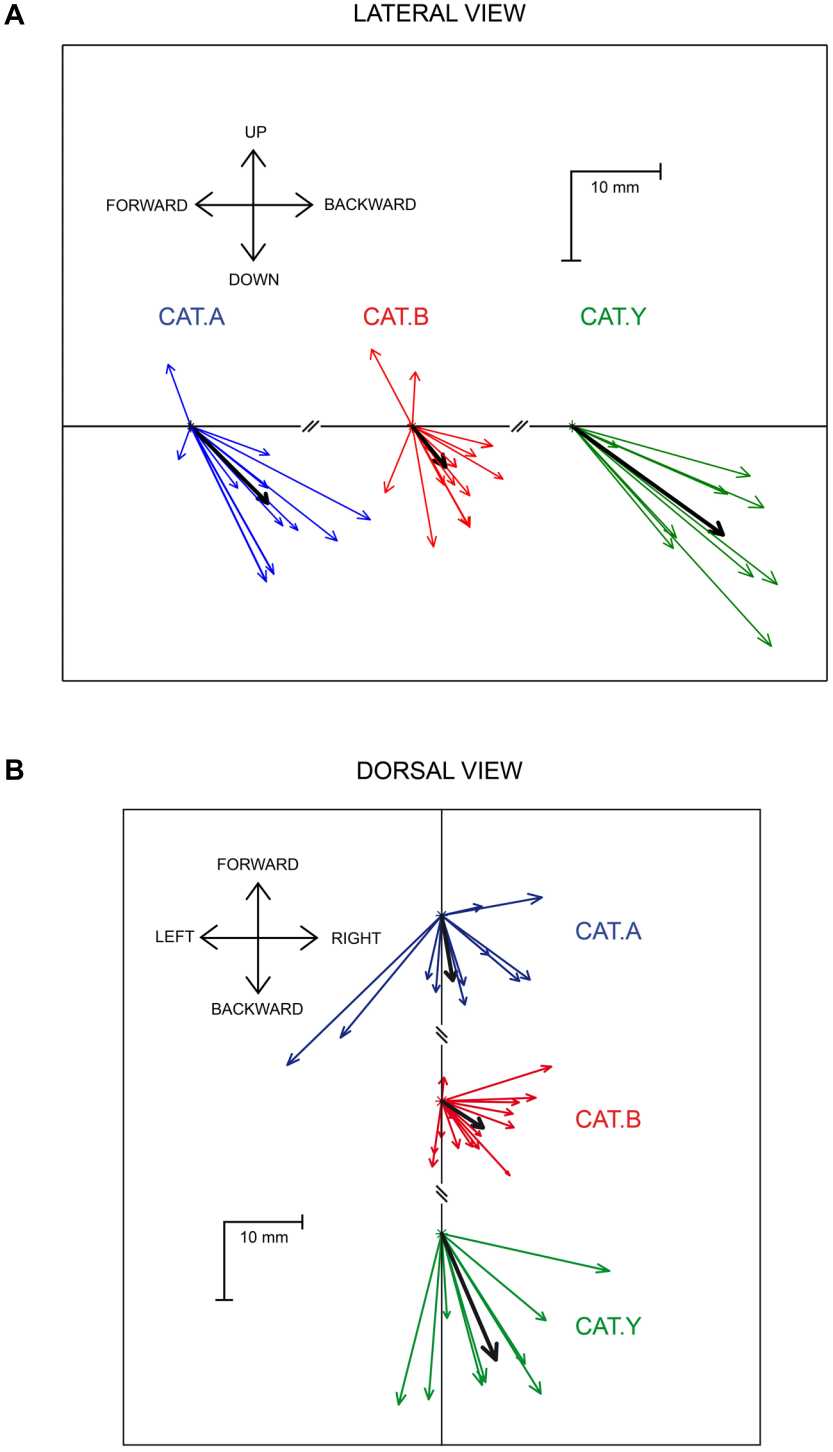
**Lateral **(A)** and dorsal **(B)** view of the perturbation vectors.** The colored arrows represent the mean perturbation vector computed for each stimulation site in the three cats. The thick black arrows represent the overall average perturbation vector for each animal. Each cat is identified by the same color as in **Figure 1**.

The average components of the perturbation vector along the three axes, X (backward), Y (lateral), and Z (downward), were first calculated on data pooled from the three cats. They all differed significantly from zero [*t*(34) > 3.59, *p* < 0.01). In addition the lateral deviation was much smaller (3.5 ± 5.8 mm) than the backward and downward deviations [-7 ± 6.8 mm; *t*(34) = 9.4, *p* < 0.001; and -8.7 ± 8.6 mm, *t*(34) = 8.3, *p* < 0.001, respectively] (see **Figure 4**). We then investigated whether the *X, Y,* and *Z* components of the perturbation vector were similar between the three cats. An ANOVA analysis pointed out that *X* and *Y* components did not significantly differ between the three cats [*F*(2,32) = 2.43, *p* > 0.05 and *F*(2,32) = 2.1, *p* > 0.05, respectively]; in contrast the *Z* component differed significantly [*F*(2,32) = 8.6, *p* = 0.001], corresponding to a larger downward deviation in cat *Y* than in cats A and B. For this reason, we normalized each of the *X, Y,* and *Z* components for each individual site with respect to the mean perturbation of each component obtained in a given animal. This normalization allowed us to compute a correlation between each normalized component and the location of the stimulation sites on the SC map. We found no significant correlation between the 3D vector components and the location of stimulated sites (rostral-caudal coordinate: *r* < 0.24, *p* > 0.17; medial-lateral coordinate: *r* < 0.20, *p* > 0.24). These results indicate that the perturbation vector amplitude and orientation did not depend on the stimulation sites on the SC map. Therefore we pooled in **Figure 4** the data gathered from all stimulation sites to represent the perturbation vectors for each cat (and the mean perturbation in each cat illustrated by the black arrow). A lateral view (**Figure 4A**) and dorsal view (**Figure 4B**) of the perturbation vectors shows that, following SC stimulation, the reaching movements were deviated both backward and downward, explaining the strong deceleration of reaching movements.

## Discussion

The current study examines the effect of electrical stimulation delivered into intermediate and deep layers of the cat SC on forelimb movements directed toward stationary targets. In agreement with our previous report (Courjon et al., 2004), we showed that SC stimulation disturbs reaching movements. However, we found that SC stimulation invariably induced a forelimb movement deviation directed downward and backward regardless of the stimulation site location on the SC map. This non-topographically organized perturbation was transient and, at the stimulation offset, the movement resumed and reached its goal with the same accuracy as in control movements.

The lack of a complete map of reach directions in the SC and the absence of congruence between the collicular oculomotor map and parameters of elicited forelimb deviations (direction and amplitude) appears puzzling with respect to the well-known topographical collicular organization for gaze displacements; this is, however, somewhat reminiscent of the broad spatial tuning reported for collicular cells controlling head movements in primates (Walton et al., 2007). This lack of topographical forelimb representation in the SC definitely questions the functional significance, and the interpretation ingrained in the literature, of these results. Indeed, previous studies aimed at investigating arm movement-related activity in the primate SC (Werner, 1993; Werner et al., 1997a; Stuphorn et al., 2000) also pointed out the lack of correspondence between the “reach cells” and SC oculomotor map. Despite this, the existence of these reach cells were presented as evidence that the SC is involved in the initiation and execution of arm movements, a view which has received further support from functional imaging studies in humans (Linzenbold and Himmelbach, 2012; Himmelbach et al., 2013). However, these observations are correlative and do not prove that the SC plays a causal role in encoding reaching movements. In an attempt to address this issue, (Philipp and Hoffmann, 2014) delivered electrical stimulation in the monkey SC, and although they showed it elicited hand or arm movements, again they failed to evidence any topographical organization, or correspondence with the SC oculomotor map. Moreover, these electrically evoked arm movements were (1) experience-dependent and thus very different between animals; (2) produced only by stimulations of the lateral SC (representing the lower part of the visual field); and (3) elicited at a depth of about 3.5 mm, i.e., the limit between the SC and underlying mesencephalic reticular formation in monkeys (Werner et al., 1997b).

Therefore, although it is indisputable that the SC exerts some influence on forelimb movements, as already suggested by early electrophysiological studies in cats (Faulkner and Hyde, 1958; Anderson et al., 1972), the recent results gathered in both cats and monkeys do not support the view that the SC might participate in initiating and/or executing visually guided arm movements. This reservation is further strengthened by a recent study showing that muscimol injections in the primate intermediate SC layers do not cause low level deficits in reaching movements but impair the target selection processing, observed only when several visual cues were competing (Song et al., 2011).

If the SC is not directly involved in movement planning or execution as suggested by the previous and current studies, then the nature of the mechanisms being disrupted by the electrical stimulation remains to be elucidated. Microstimulation could have led to a covert shift of attention to a spatial location distant from the reach target (Cavanaugh and Wurtz, 2004; Müller et al., 2005), leading to a temporary disruption of the on-going movement, which would then resume after the stimulation offset, when spatial attention shifts back to the target. Attentional shifts away from the target should lead to identical consequences on reaching movements whatever the location of the stimulation site on the SC map, in accordance with our findings. However, the latency of the effects seen in our study, occurring 40–60 ms after SC stimulation, appears inconsistent with the interpretation of a visual information alteration leading to online adjustment of the reaching movement. Indeed, the transformation of the visual information about target location into reaching movement parameters in the parietal cortex requires about 150 ms in monkeys, to which should be added 50–100 ms before movement execution (Archambault et al., 2009). The “shift of attention hypothesis” thus predicts that the latency of movement perturbations should be larger than that we found in the current study, suggesting that SC stimulation disturbed reaching movements at a later stage of processing.

It could be proposed that this stage is the target selection process, in accordance with the view of Song et al. (2011) collaborators. These authors showed recently that most cells in the intermediate layer of the primate SC, which exhibit prolonged activation during delayed-saccade tasks, also signal the spatial location of reach targets (Song and McPeek, 2015). In accordance with our results, this “target selection hypothesis” predicts that, when no visual target is present at the retinotopic location stimulated on the SC map the stimulation should lead to a disruption of the target selection process and to an interruption of the movement. Furthermore, the “target selection hypothesis” also predicts that if an alternative visual reach target were presented at the spatial location corresponding to the stimulated site on the SC map, the stimulation should lead to a movement reprogramming toward this alternative target, rather than a movement interruption. This behavior remains to be explored in future experiments but is consistent with the effect of SC inactivation during motion discrimination tasks, which leads the animal to follow accurately the motion direction of the wrong stimulus, indicating a deficit in target selection (Lovejoy and Krauzlis, 2010; Zénon and Krauzlis, 2012).

Another possible explanation of our finding is that the orienting behavior (eye, head or even body turning) potentially caused by SC stimulation could have created the need to stabilize the posture (Midgley and Tees, 1981) and therefore to suspend the reaching movement for returning to a four-legged steady position. This might explain why we always observed the same reach deviation following SC stimulation, with an evoked movement reminiscent of a limb returning to the ground.

Additional experiments will be necessary to investigate further the role of the SC in controlling arm movements and to tease apart these different hypotheses.

## Conflict of Interest Statement

The authors declare that the research was conducted in the absence of any commercial or financial relationships that could be construed as a potential conflict of interest.
